# Relapsing Nasal Septal Leishmaniasis: Lessons in Long‐Term Multidisciplinary Management Case Report

**DOI:** 10.1155/crdi/1784961

**Published:** 2026-06-17

**Authors:** Emily Ehsan, Ellie Staab, Katie Geelan-Hansen, Richard Hankins

**Affiliations:** ^1^ College of Medicine, University of Nebraska Medical Center, Omaha, Nebraska, USA, unmc.edu; ^2^ Department of Otolaryngology-Head and Neck Surgery, University of Nebraska Medical Center, Omaha, Nebraska, USA, unmc.edu; ^3^ Division of Infectious Diseases, Department of Internal Medicine, University of Nebraska Medical Center, Omaha, Nebraska, USA, unmc.edu

**Keywords:** amphotericin B, case report, *Leishmania braziliensis*, *Leishmania braziliensis,* mucosal leishmaniasis, recurrence

## Abstract

**Introduction:**

Mucosal leishmaniasis (ML) is a rare, destructive, and recurrent protozoal infection caused by *Leishmania* species, most commonly *Leishmania braziliensis*. ML can develop months to years after initial cutaneous leishmaniasis (CL) and may lead to severe mucosal destruction if untreated.

**Case Presentation:**

We present a case of a 48‐year‐old healthy male from Venezuela with prior CL treated with intravenous meglumine antimoniate who subsequently developed progressive nasal symptoms consistent with ML. Initial therapy with oral fluconazole was ineffective, but treatment with miltefosine led to symptom resolution. The patient underwent four‐staged reconstructive surgeries with ENT. Thirty‐eight months later, the patient developed recurrent symptoms, and biopsy confirmed infection with *L. braziliensis*. He was subsequently treated with intravenous amphotericin B, which led to clinical improvement.

**Discussion:**

This case highlights the relapsing nature of ML, its potential for extensive mucosal damage, and the importance of a structured, long‐term care plan. This case in particular is unique given the extended delay before recurrence following miltefosine therapy. The high recurrence rate of *L. braziliensis* infections underscores the importance of vigilant long‐term follow‐up and consideration of systemic therapy. Multidisciplinary management is often necessary to optimize outcomes.

**Conclusion:**

Early recognition of ML, timely systemic therapy, structured multidisciplinary care, and adherence to a long‐term surveillance plan are critical to prevent mucosal destruction and manage relapses effectively. Extended surveillance may be warranted to detect late relapses in patients treated with newer or more effective agents.

## 1. Introduction

Leishmaniasis is a protozoal infection transmitted by female sand fly vectors. Clinical manifestations can be cutaneous, mucosal, or visceral, and severity varies based on parasite virulence and host immune response. It most commonly presents as localized cutaneous leishmaniasis (CL). Leishmaniasis lesions are generally chronic, painless, and tend to cluster on exposed skin. Patients may not recall a sand fly bite even if they have traveled to endemic regions.

Mucosal leishmaniasis (ML), a less common presentation of the protozoal infection, occurs in both the New and Old World. New World ML is found in South and Central America and is caused by *Leishmania (Viannia)braziliensis*, *L.(V.) guyanensis*, *L.(V.) panamensis*, and *Leishmania (Leishmania) amanzonesis*. Old World ML species are more commonly seen in immunocompromised hosts and include *L.(L.) infantum* and *L.(L.) aethiopica* [1]. ML can result in mucosal destruction with symptoms such as persistent nasal congestion, mucosal bleeding, increased secretions, sloughing of necrotic tissue, inflammation, deformity, and pain. Mucosal involvement commonly affects the nose, nasal septum, or mouth but can extend to the cheeks, pharynx, palate, epiglottis, larynx, trachea, and genitalia [1].

Definitive diagnosis relies on sampling active lesions with culture, histopathology, and polymerase chain reaction confirming infection [2]. Treatment should be individualized based on *Leishmania* species and disease severity. ML is more difficult to treat than CL, especially when advanced [3]. Otolaryngologic evaluation is necessary prior to therapy to assess mucosal extent. Therapeutic options include parenteral or intramuscular pentavalent antimonials, amphotericin B (including liposomal formulations), and miltefosine [4]. Liposomal amphotericin B and miltefosine are both considered first‐line treatments for ML. Due to recurrence risk, patients should be monitored for six to 12 months after completion of treatment, and at least two years if there is mucosal involvement. Definitive clinical cure of ML is considered re‐epithelialization of mucosal ulcers and resolved inflammation of lesions without relapse 12 months posttreatment.

ML is ultimately a severe and destructive disease presentation that is difficult to treat. We present a case of recurrent leishmaniasis with severe nasal malformation, outlining our medical management and surgical reconstruction.

## 2. Case Presentation

**FIGURE 1:**
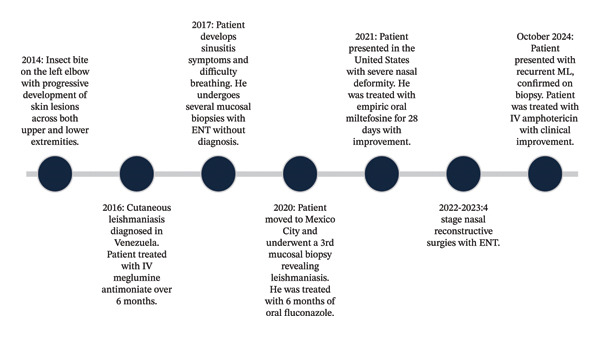
Timeline of major clinical events including symptom onset and therapeutic interventions.

A 48‐year‐old healthy male from South America presented with progressive sinusitis symptoms, difficulty breathing, intranasal crusting, and facial erythema. This patient was originally treated for CL in 2016 in Venezuela with 68 doses of intravenous (IV) meglumine antimoniate (Glucantime) over the course of 6 months (Figure 1). He recalled an insect bite in 2014 on his left elbow followed by several skin lesions, which was considered the likely site of *Leishmania* exposure. Posttreatment with meglumine antimoniate, the patient had residual scarring of his elbow but otherwise felt well. Six to 12 months after completing treatment, he developed sinusitis symptoms and difficulty breathing. The patient had difficulty obtaining a diagnosis in Venezuela with multiple nondiagnostic mucosal biopsies. He immigrated to Mexico City in 2020 where a third biopsy revealed leishmaniasis. He was placed on oral fluconazole, initially 50 mg and later increased to 150 mg.

**FIGURE 2:**
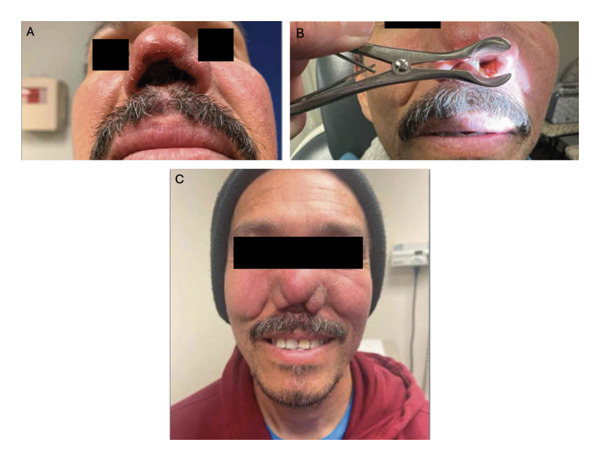
Clinical course of nasal septum destruction and reconstructive management. (A) Initial presentation examination demonstrating complete erosion of the nasal septum and columella. (B) Postseptorhinoplasty with costal cartilage graft, scar revision, division and inset of bilateral melolabial flaps, and facial plastic reconstruction including rhinoplasty revision and ear cartilage graft. (C) Post most recent treatment with IV amphotericin.

The patient immigrated to the United States in the summer of 2021 and presented for continued sinusitis symptoms. Physical exam revealed absent columella and anterior septum, erythematous and thickened nasal mucosa with ulceration, and erythematous thickening extending to the bilateral cheeks (Figure 2A). Punch biopsies of the upper lip skin and right nasal mucosal floor revealed granulomatous inflammation, but no microorganisms on GMS, acid‐fast, or Giemsa stains. PCR testing with the CDC was unavailable due to the COVID pandemic. The patient had been on oral fluconazole for approximately six months with minimal improvement, so fluconazole was discontinued, and the patient was started on a 28‐day course of daily oral miltefosine 50 mg in August 2021 [5]. Posttreatment with miltefosine, the facial erythema, sinus swelling, and shortness of breath resolved, and repeat biopsy at 6 months showed resolution of granulomatous inflammation.

Head and neck surgery performed a series of operations to repair the nasal septum and restore function and cosmesis. Between 2022 and 2023, the patient underwent staged surgical reconstruction including septorhinoplasty with costal cartilage graft, scar revision, bilateral melolabial flap inset, and final rhinoplasty revision with ear cartilage graft, which restored both function and appearance.

In October 2024, the patient again experienced recurrent sinusitis. The patient’s recurrent symptoms mirrored those of the initial presentation (Figure 2B), although he had no repeat exposure to *Leishmania*. Biopsy sent to the CDC‐confirmed *L. braziliensis* infection. While the patient demonstrated a response to miltefosine, treatment with IV amphotericin B 3 mg/kg on Days 1–5, 10, and 21 was chosen due to his history of recurrence. One month following treatment, the patient had significant clinical response to treatment with resolution of his facial erythema and sinusitis, and improvement in his breathing (Figure 2C). The patient is seen in clinic every 4 months, and there have been no signs or symptoms of recurrence to date. Written consent was obtained from the patient for this case report.

## 3. Discussion

This case illustrates the complex and relapsing course of ML, a less common but more destructive manifestation of *Leishmania* infection than CL. Although ML is rare, it can cause progressive mucosal destruction leading to significant functional impairment and facial disfigurement. Research suggests that 95% of ML recurrence occurs within the 36 months of initial infection [6]. In this patient, recurrence occurred 38 months after treatment, falling into the rare 5% of patients with delayed recurrence. And in our patient, prior therapy resulted in nasal septum and columella destruction, chronic nasal obstruction, and facial deformity requiring staged reconstructive surgery. This presentation highlights the high morbidity associated with recurrent ML and underscores the long‐term consequences of recurrence following miltefosine treatment of *L. braziliensis*.

ML is an uncommon disease, and current IDSA guidelines provide no specific recommendations for recurrent disease, leaving management to clinical judgment and limited published experience [2]. Recurrence rates for ML are reported at 15%–20%, particularly with *L. braziliensis*, which exhibits a strong predilection for mucosal involvement and the capacity for chronic or latent infection [7, 8]. The mechanisms underlying mucosal tropism and relapse include immune evasion, persistent tissue reservoirs, and local inflammatory responses that promote progressive tissue destruction [9]. In this patient, initial treatment with oral miltefosine was followed by relapse more than 3 years later, confirmed by biopsy and PCR, demonstrating the challenges of achieving durable remission and adding novel data on the long‐term risk of recurrence following miltefosine therapy.

Recurrent ML presents significant therapeutic challenges. While miltefosine is widely used as first‐line therapy due to its oral administration and efficacy, relapse often necessitates alternative agents. In this case, amphotericin B was selected based on prior treatment failure, with liposomal amphotericin B preferred for its safety profile and proven efficacy against *Leishmania* species [10]. This therapeutic course emphasizes the importance of individualized treatment strategies guided by prior therapies, disease severity, and recurrence risk.

Optimizing outcomes in recurrent ML requires close surveillance and multidisciplinary care. Routine otolaryngologic evaluations are essential as mucosal relapse can progress silently until irreversible structural damage occurs. Collaboration between infectious disease specialists, dermatologists, and surgical teams is critical for both infection control and management of functional and cosmetic sequelae. In this patient, coordinated care enabled disease control and successful nasal reconstruction with staged procedures, including costal cartilage grafts and soft tissue flaps.

This case underscores several key principles in ML management: the high risk of recurrence, the need for multiple systemic therapies in recurrent cases, staged surgical reconstruction and the critical role of coordinated, long‐term multidisciplinary follow‐up, and the unclear timing of recurrence following miltefosine therapy. As a single‐patient case report, the findings are not generalizable, and the inability to obtain confirmatory PCR testing during the initial U.S. evaluation due to pandemic‐related limitations represents a diagnostic constraint. Importantly, this case highlights that, following miltefosine therapy, recurrence may occur well beyond the standard 2‐year monitoring period. While the literature has shown that most recurrence cases occur with 36 months of initial treatment, this based mostly on patients treated with Glucantime [6]. The timing of *L. braziliensis* recurrence after miltefosine therapy remains unclear. Consequently, extended surveillance may be warranted to detect late relapses in patients treated with newer or more effective agents. Despite these limitations, this case contributes valuable insight into the relapsing nature and multidisciplinary management of ML. Monitoring for at least 2 years is particularly important in *L. braziliensis* infection given its propensity for latent and mucosal disease. By demonstrating the morbidity, therapeutic complexity, and necessity of integrated care, this case provides perspective on recurrent ML and reinforces the importance of surveillance in patients even after apparent clinical remission.

## 4. Conclusion

ML due *to Leishmania braziliensis* is a rare but destructive complication that may emerge months to years after initial cutaneous infection and carries a relapse risk of up to 20%. Clinicians should maintain a high index of suspicion in patients with prior CL who develop new sinonasal or oropharyngeal symptoms, even after apparent treatment success. Given the risk of progressive structural damage and functional impairment, optimal management requires coordinated long‐term follow‐up between infectious disease specialists and head and neck surgeons. Early recognition of recurrence and multidisciplinary care are essential to prevent irreversible morbidity in patients with *L. braziliensis* infection. As relapse can occur despite appropriate first‐line therapy, patients require prolonged clinical surveillance with careful endoscopic examination of mucosal sites. Treatment failure with oral agents such as miltefosine should prompt early consideration of IV amphotericin B. Extended surveillance may be warranted to detect late relapses in patients treated with newer or more effective agents.

## Author Contributions

Emily Ehsan and Ellie Staab did patient review and case writing, Katie Geelan‐Hansen and Richard Hankins reviewed the manuscript, Richard Hankins oversaw the case study process.

## Funding

No funding was received for this manuscript.

## Disclosure

All authors have reviewed and approved the final version of the manuscript.

## Ethics Statement

This case study was IRB‐exempt, and consent was formally obtained from patient for publication.

## Conflicts of Interest

The authors declare no conflicts of interest.

## Data Availability

No datasets were generated or analyzed during the current study.
